# Nitrogen enrichment reduces parasitism in an annual hemiparasite

**DOI:** 10.1002/ajb2.70101

**Published:** 2025-09-18

**Authors:** Jasmine Taite, Paul D. Nabity

**Affiliations:** ^1^ School of BioSciences University of Melbourne Melbourne 3010 Victoria Australia

**Keywords:** *Castilleja exserta*, climate change, haustoria, *Nasella lepida*, *Nasella pulchra*, Orobanchaceae, plant parasite, Poaceae, pollution, root behavior

## Abstract

**Premise:**

Anthropogenic activities such as fossil fuel combustion and synthetic fertilizer synthesis have resulted in nitrogen (N) deposition and elevated N availability in ecosystems. Many parasitic plants are adapted to low N environments and have evolved mechanisms to sequester water, N, and other limiting nutrients from hosts. Anthropogenic N deposition may perturb these host–parasite interactions, thereby altering nutrient cycling and ultimately reducing biodiversity.

**Methods:**

To test how N enrichment affects the incidence and degree of plant parasitism, we assayed host–parasite performance and in vitro root growth under supplemental N levels representative of urban areas experiencing N deposition. We focused on the annual hemiparasite *Castilleja exserta* and two co‐occurring host species, *Nasella lepida* and *N. pulchra*.

**Results:**

Elevated N decreased haustoria formation and host‐seeking behavior by roots but did not affect growth, suggesting N enrichment may reduce parasitism without associated loss of parasite biomass. We confirmed that parasitism decreased host performance and that the degree of parasitism was positively related to host biomass and earlier flowering. We also found *N. lepida* may be preferred over *N. pulchra* as a host.

**Conclusions:**

These results indicate that N pollution altered parasitism in *C. exserta* and highlight the far‐reaching ecological effects of N pollution on host–parasite interactions within plant communities.

Shifts in climate impact ecosystem function by altering species interactions (Parmesan and Hanley, 2015; Franklin et al., 2016), including host–parasite interactions that have an outsized effect on community structure (Harvey et al., 2017; Frainer et al., 2018). However, knowledge on how parasitic plants respond to rapid environmental change remains limited. Historically, select parasitic genera (e.g., *Rhinanthus* spp.) have been directly assessed for how elevated carbon dioxide (CO_2_) alters individual performance and host interactions, confirming CO_2_ stimulates parasite growth but has different indirect effects on the parasite depending on how nutrients are acquired through the host (Hwangbo et al., 2003; Phoenix and Press, 2005). Recent attention has focused on how shifts in precipitation and temperature alter habitat and host range suitability, showing reduced spatial overlap of parasites and hosts (e.g., Lu et al., 2022). Additionally, elevated temperature can increase parasite biomass and host connectivity and thus negate any stimulation in host growth (Nyléhn and Totland, 1999; Rafferty et al., 2019). Because parasitic plants range from entirely heterotrophic to hemiparasitic (i.e., with varying levels of autotrophic photosynthesis) and directly influence key ecosystem processes such as decomposition and nutrient cycling through nutrient sequestration (Bardgett et al., 2006; Spasojevic and Suding, 2011), these specialized plants can provide relevant insight for predicting plant growth and use of resources under environmental change.

Anthropogenically driven climate change has rapidly influenced temperatures and water availability, but the drivers of these effects (e.g., combustion emissions such as NO*x*) has also altered nutrient availability through nitrogen (N) deposition. For areas downwind of large metropolitan and agricultural centers, increased N loading in soils can have cascading effects. For example, N deposition near urban areas in the Southwest United States increased annual biomass/productivity, favoring invasive or weedy species that, in turn, altered fire incidence and intensity (Fenn et al., 2003; Valliere et al., 2017). Enhanced N also reduced mycorrhizal competency (Allen et al., 2014, 2016) and species network complexity (Sun et al., 2021) and can decrease species richness when above N saturation, especially in low‐nutrient ecosystems (Li et al., 2023), ultimately reducing ecosystem stability. Because N is a major nutrient limiting plant growth, low‐nutrient environments likely promote the evolution of diverse acquisition strategies that range from mycorrhizal and nodule‐forming mutualisms to gradients of parasitism and intrinsic N‐use efficiency (Moreau et al., 2019; Tao et al., 2019; Zhang et al., 2023). Thus, additional information on how N deposition perturbs sensitive, keystone interactions is needed to predict community persistence as species experience rapid environmental change.

For parasitic plants, N availability alters persistence through changes in both host and parasite physiology. Typically, plant parasites attach to host vascular tissues through the formation of haustoria, then rely on transpiratory efflux to drive acquisition of resources (establishing the N–parasitism hypothesis; Ehleringer et al., 1985). For hemiparasitic species, increasing host N can offset the fitness costs of parasitism, possibly because of reduced transpiratory flux that directly alters parasite N uptake and demand (Marshall et al., 1994; Ossa et al., 2021; Zhang et al., 2023). For root hemiparasites, increasing soil or host N may reduce (Cechin and Press, 1993; Irving et al., 2019), enhance parasite growth (Frederica and Irving, 2024), or have no effect on (Sinebo and Drennan, 2001), illustrating the context dependency of N uptake and transport for hemiparasites. Additionally, when the host fixes N via rhizobia, the strength of parasitism varies depending on the interacting species (Cirocco et al., 2017). Because plant parasites independently evolved across clades, lineage‐specific differences may underlie these variable effects of N on host–parasite interactions. Therefore, additional sampling of understudied taxa will fill key gaps and reveal more generalizable patterns. Recently, haustoria formation was shown to decline with increasing N through host abscisic acid synthesis and signaling, key regulators of transpiratory efflux. Because this decline was found in unrelated root hemiparasites *Phtheirospermum japonicum* and *Striga hermonthica*, a general mechanism for N to modulate parasite intensity via transpiration and related signaling may exist (Kokla et al., 2022). If this hypothesis holds for all plant parasites, we predict changes in environmental N should directly alter haustoria formation and overall parasitic plant function in natural communities.

Here we focused on the interaction between *Castilleja exserta* (Heller) Chuang & Heckard (Orobanchaceae), a hemiparasitic forb, and its co‐occurring *Nassella* host species when exposed to naturally occurring and emission‐deposited levels of N found adjacent to urban centers. We examined the effects of N on the hemiparasite and hosts alone and when paired during parasitism. We assessed greenhouse performance and in vitro germination to address the following questions: How does N enhancement under N deposition levels alter performance of a hemiparasite and its host species? How does N alter host‐seeking behavior and colonization (via haustoria) of a root hemiparasite?

## MATERIALS AND METHODS

### Plant and soil material

*Castilleja exserta*, commonly known as purple owl's clover, is an annual root hemiparasite found in sandy, well‐drained soils across ecoregions from Northern to Baja California and inland into Arizona and New Mexico. A spring annual, it blooms from March to July and is an important host plant for a range of butterflies, including the Bay checkerspot butterfly, *Euphydryas editha bayensis*, which is listed as threatened in California. Anecdotal information lists several co‐occurring plant species, including those in the genus *Nassella* (syn. *Stipa*), as *C. exserta* hosts, but root attachment remains understudied for *C. exserta*. *Nassella lepida* (Hitchc.) Barkworth and *Nassella pulchra* (Hitchc.) Barkworth (Poaceae) are perennial bunch grasses native to southern California that grow predominantly through winter andspring and become dormant in and co‐occur with *C. exserta* (California Native Plant Society, 2025) on sites experiencing increased N deposition (Allen et al., 2014). To establish N treatment concentrations, we surveyed the literature and identified locations in southern California (CA) where all species were present and N deposition rates were known. In the last decade, some sites received up to 30 kg N ha^−1^ year^−1^ via deposition, resulting in soil concentrations of 10–40 μg/g (Allen et al., 2014). These soil concentrations are likely to reflect current levels; while pollution controls have reduced N deposition rates in the United States, the Southwest had the slowest decline (~0.02 kg N ha^−1^ year^−1^) during 2002–2017, with the average rates of decline lower from 2010–2017 compared to 2002–2009 (Benish et al., 2022). These data suggest soils within our species' ranges have had elevated N over longer periods; however, soil N may also increase rapidly with adjacent wildfires (Campbell et al., 2022). In the absence of real‐time rates of deposition, we selected rates typical of our study region in 2014 for our experiments. All plants used in this study were grown from locally sourced seed purchased in 2022 (Theodore Payne Foundation; Sun Valley, CA, USA).

### Performance assay

To assess whether N levels associated with deposition altered host–parasite interactions, we assayed performance/growth of host–parasite pairs (pairing treatments) under naturally (low) N levels found across the range of *C. exserta* and elevated (high) N found in areas under urban N deposition. All plants were grown in a greenhouse with ambient spring photoperiod (increasing from 10 h light:14 h dark to 14 h light:10 h dark) February through June and constant day and night temperatures (24 ± 3°C day, 27 ± 3°C night). Seeds were planted in 1.9‐L pots filled with autoclaved soil (UC Soil Mix III: https://agops.ucr.edu/soil-mixing#uc-soil-mix-iii) with the following pairing treatments: *C. exserta* paired with *N. lepida* (C + L), *C. exserta* paired with *N. pulchra* (C + P), *C. exserta* alone (C), *N. lepida* alone (L), and *N. pulchra* alone (P) and thinned to one seedling per species after germination (Appendix S1: Figure S1). For our N treatments, pots were randomly assigned an ambient N amendment of 13 µg/g N typical for Southern CA soils, or an elevated N level of 40 µg/g. The elevated N treatment was created by watering once with 0.177 g NH_4_NO_3_ per pot in trays (8 pots per tray) to prevent N leaching/runoff. All pots within a tray received the same N treatment to avoid leaching between pots. Pots in trays were watered as needed throughout the experiment, and each condition was applied to 20 pots across trays. Plants were harvested after flowering and washed with a fine‐mist water stream to loosen soil from roots. This gentle washing preserved haustoria connections and allowed for separation of above‐ and belowground biomass. For root samples with haustoria (C, C + L, and C + P), roots were immersed in water and stored at 4°C until counting and separation with a STEMI 508 stereomicroscope (Zeiss, Jena, Germany). A gradient was established to count the haustoria and distinguish between visible and ambiguous connections (Appendix S1: Figure S2 *)*. After the counting, roots were transferred to envelopes and dried with the aboveground biomass at 50°C, then weighed. For all plants, above‐ and belowground biomass was recorded, and for *C. exserta*, we also recorded the date of first flowering and quantified stem height and number and haustoria number.

### In vitro bioassay

To assess whether root growth was directional toward hosts and whether any directional growth was altered by N, both *C. exserta* and the *Nassella* hosts were grown beside each other in nutrient‐modified agar as described by Kokla et al. (2022). Initially, *C. exserta* seeds were treated with 5% v/v bleach for 5 min; *N. lepida* and *N. pulchra* seeds were treated with 10% v/v bleach for 10 min. Sterilized seeds were set in petri dishes containing 0.7% w/v agar at N concentrations of 0 mM, 10 mM (elevated N level) and 25 mM (high N level) by adding NH_3_NO_4_ to the medium—the equivalent of 0, 40, and 100 μg N per g soil (2.5× higher than at depositional sites). Seeds of *C. exserta* were placed either 0.5, 1, or 2 cm from a focal target seed (*N. lepida*, *N. pulchra*, or another *C. exserta*) in 7‐cm petri dishes, and at least three replicates of each nutrient × distance treatment were set up. After 30 days, any dishes with germinated seeds of *C. exserta* were photographed, and ImageJ was used to measure root length toward or away from the focal seed and determine whether the root changed direction (turned; see Appendix S1: Figure S3). Previous studies have shown image analysis of roots within nutrient modified agar is suitable for host choice experiments (Sandner et al., 2022). Total attractiveness of a focal seed was determined by the net summation of root length toward (+) and away (‐).

### Statistical analyses

For performance assays, we fit linear mixed‐effects models (LMMs) for the total, aboveground, and belowground biomass response variables. Each model included N treatment and pairing treatment (with or without host) and their interaction as predictors, with tray as a random effect. We included interaction terms to understand how host presence and N amendment interact, given both are sources of N. For all models, fit was determined by a likelihood ratio test comparing log‐likelihood scores of nested models. When the interaction terms were not significant and did not improve the model fit, we removed them to reduce model complexity. We fit models separately for *C. exserta* and the hosts. We also fit all models for paired‐only data (*C. exserta* and *Nassella* host pairs) with N treatment and host species as predictors to understand the effect of N on paired plant growth and relative to species identity. In all LMMs, when the random effects were negligible, we dropped the term and fit linear models (LMs).

To assess how treatments affected haustoria formation, we fit a generalized linear mixed‐effects model (GLMM) with a Poisson error distribution. In this model, haustoria count was the response; N treatment, host presence, and their interactions were the predictors; and tray was a random effect. Because gains in biomass and haustoria are gains in mass, we included total biomass as a covariate. To avoid variance inflation, we also tested for collinearity among interactions and removed variance inflation factors (VIF) > 7. In all GLMMs, when the random effects were negligible, we dropped the term and fit generalized linear models (GLMs) with a Poisson error distribution. We also fit GLMMs for paired‐only data (*C. exserta* and *Nassella* host pairs) with N treatment, host species identity, total biomass, and the interaction as predictors. We removed interactions as described above. In all GLMMs, when the random effects were negligible, we dropped the term and fit GLMs. If interaction terms were significant, we conducted post hoc (Tukey) tests among groups. We compared model fit by likelihood ratio tests and report only the best‐fitting model as previously described.

To assess how our treatments affected parasite flowering time, we fitted a mixed effects Cox proportional hazards regression model (https://github.com/therneau/survival; library coxme) suitable for analyzing time to an event. We assessed whether time to flowering varied with total biomass, N treatment, host presence, and their interactions as the predictors and tray identity as a random effect. Interaction terms were removed when not significant or when VIF > 7 indicated collinearity. Hazard ratios or rate at which the event is likely to occur are reported. To assess stem height and number, we fit LMM and GLM models for continuous and count data respectively, as described previously.

To analyze data from our bioassay of root growth, we fit LMMs with total parasite root length and attraction to the focal seed as the responses and distance between seeds, N treatment (zero, elevated, high), and pairing treatment as predictors. We included dish as a random effect but removed the term when negligible and refit with a LM. We also used a *χ* ^2^ test with Yates continuity correction to test whether *C. exserta* roots turned more or less than expected by chance (1:1 toward to away) relative to the other or target individual within the same dish.

All analyses were conducted in R (version 4.5.1; R Core Team, 2025). In all cases, we compared the fit of nested models with likelihood ratio tests by ANOVA and report only the best‐fitting models. The reference level for each predictor was determined alphabetically (e.g., *N. pulchra* – *N. lepida* or host:yes – host:no) or numerically (e.g., 40‐0, 100‐0), except that N level was re‐leveled to contrast from high – low. Finally, we used the emmeans function in R (Lenth, 2017) to conduct (Tukey) post hoc tests for pairwise differences when interactions were significant. We also tested whether outliers affected the model fit for total haustoria by ranking the largest residuals by Cook's distance and iteratively removing points >1 that also contained hat values above the expected threshold (2 × model parameters/*N*). This determined the degree of leverage each point had over the model.

## RESULTS

**Figure 1:**
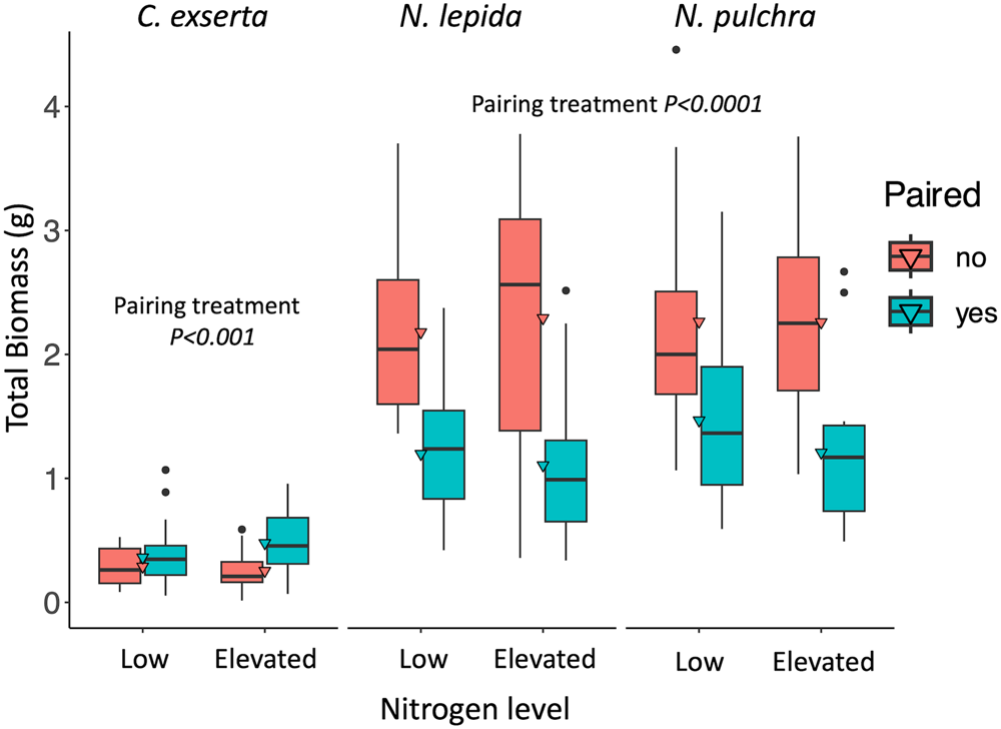
Effects of parasitism and N amendment on biomass for *Castilleja exserta*, *Nassella lepida*, and *N. pulchra* grown alone or as a host–parasite pair. Box plot shows range between 1st and 3rd quartiles; triangles: mean, bars: median, and circles: outlier.

**Figure 2:**
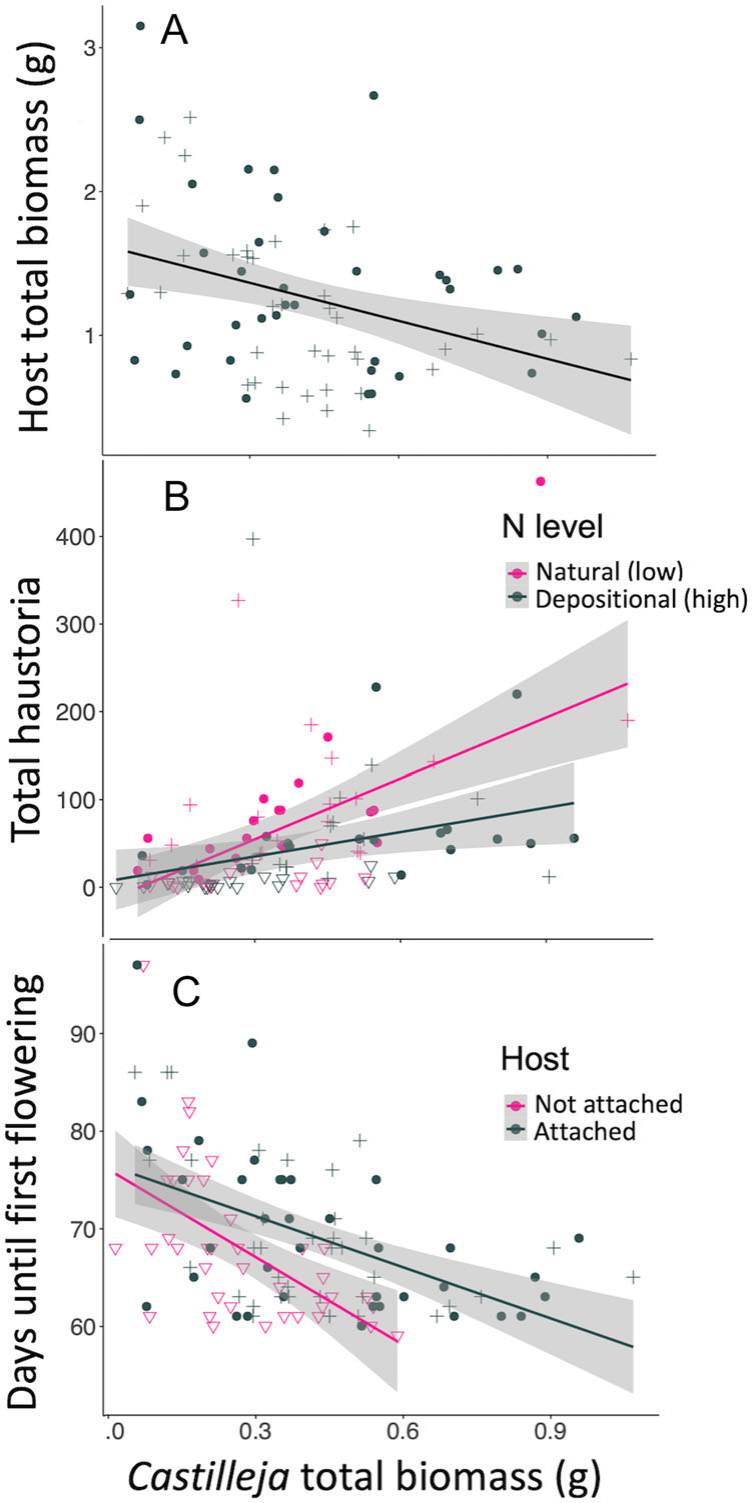
Relationship between *Castilleja exserta* biomass and (A) *Nassella* host biomass, (B) total number of haustoria formed in ambient vs elevated depositional N levels, and (C) the time to first flowering. Circles: *N. pulchra*; pluses: *N. lepida*; triangles: *C. exserta* alone.

In greenhouse performance trials, we found no effect of N treatment across all plants but pairing with a host increased parasite biomass (by 55% for total and 58% for aboveground) and decreased host biomass ~50% (Table 1, Figure 1; Appendix S2: Table S1). When host–parasite pairs were examined, N increased parasite biomass ~28% in total and for both aboveground and belowground biomass, but did not alter host species biomass (Table 2). Species identity did not alter parasite biomass but *N. pulchra* produced more belowground biomass than *N. lepida* when grown with *C. exserta*. Host biomass declined with increasing parasite biomass—0.88 g of host lost per 1 g of parasite gained—but N amendment did not alter this effect (Table 1, Figure 2A). The root mass fraction (belowground to aboveground biomass) did not vary with N but decreased 33% when a host was attached (Table 1).

**Table 1:** Best‐fitting models for greenhouse performance assays and in vitro bioassays. Predictor levels were oriented N level: high or low and Host treatment: yes or no.

Assay	Species	Response	Model	Distribution	Predictor	Estimate ± SE	*t* or *z*	*P*	*N*
Performance	*C. exserta*	Total biomass	LM	Gaussian	N: high	0.07 ± 0.04	1.69	0.094	113
					Host: yes	0.15 ± 0.04	3.69	**<0.001**	
		Aboveground biomass	LM	Gaussian	N: high	0.06 ± 0.04	1.74	0.085	113
					Host: yes	0.15 ± 0.04	3.86	**<0.001**	
		Belowground biomass	LMM	Gaussian	N: high	0.002 ± 0.002	1.14	0.258	112
					Host: yes	–0.001 ± 0.002	–0.38	0.706	
		Root mass fraction	GLMM	Beta	N: high	–0.002 ± 0.14	–0.02	0.988	112
					Host: yes	–0.32 ± 0.14	–2.19	**0.029**	
		Total haustoria	GLMM	Poisson	N: high	0.23 ± 0.06	3.72	**<0.0002**	109
					Host: yes	2.08 ± 0.06	33.73	**<0.0001**	
					Total biomass	1.82 ± 0.07	24.31	**<0.0001**	
					N * Total biomass	–1.40 ± 0.11	–11.93	**<0.0001**	
		Time to flower	GLMM‐CoxPH	Poisson	N: high	0.07 ± 0.21	0.35	0.720	112
					Host: yes	0.18 ± 0.45	0.4	0.690	
					Total biomass	6.32 ± 1.29	4.91	**<0.0001**	
					Host * Total biomass	–3.66 ± 1.33	–2.74	**0.006**	
	*Nasella* spp.	Total biomass	LM	Gaussian	N: high	–0.06 ± 0.11	–0.49	0.630	157
					Host: yes	–1.01 ± 0.11	–8.80	**<0.0001**	
		Aboveground biomass	LM	Gaussian	N: high	–0.03 ± 0.07	–0.50	0.618	157
					Host: yes	–0.63 ± 0.07	–8.96	**<0.0001**	
		Belowground biomass	LMM	Gaussian	N: high	–0.02 ± 0.05	–0.38	0.710	157
					Host: yes	–0.38 ± 0.05	–7.05	**<0.0001**	
Lab Bioassay	*C. exserta*	Total length	LMM	Gaussian	N: elevated	–0.48 ± 0.16	–3.01	**<0.01**	146
					N: high	–0.67 ± 0.16	–4.26	**<0.0001**	
					Species: *N. lepida*	0.05 ± 0.15	0.35	0.728	
					Species: *N. pulchra*	–0.37 ± 0.16	–2.30	**0.025**	
		Attraction	LMM	Gaussian	N: elevated	0.21 ± 0.21	0.98	0.337	146
					N: high	0.29 ± 0.21	1.40	0.160	
					Species: *N. lepida*	0.88 ± 0.21	4.20	**<0.0001**	
					Species: *N. pulchra*	0.51 ± 0.22	2.30	**0.021**	

**Table 2:** Best‐fitting models for greenhouse assays for only parasite‐host pairings. Predictor levels were oriented N level: high or low and Host species: *N. pulchra* or *N. lepida*.

Assay	Species	Response	Model	Distribution	Predictor	Estimate ± SE	*t* or *z*	*P*	*N*
Performance	Paired *C. exserta*	Total biomass	LM	Gaussian	N: high	0.11 ± 0.05	2.2	**0.031**	75
					Host: *N. pulchra*	0.02 ± 0.05	0.35	0.720	
		Aboveground biomass	LM	Gaussian	N: high	0.11 ± 0.05	2.26	**0.027**	78
					Host: *N. pulchra*	0.003 ± 0.05	0.07	0.950	
		Belowground biomass	LMM	Gaussian	N: high	0.004 ± 0.002	2.02	**0.047**	74
					Host: *N. pulchra*	–0.001 ± 0.002	–0.05	0.960	
		Root mass fraction	GLMM	Beta	N: high	0.02 ± 0.16	0.14	0.890	74
					Host: *N. pulchra*	–0.05 ± 0.16	–0.29	0.770	
		Total haustoria	GLMM	Poisson	N: high	–0.42 ± 0.03	–14.60	**<0.0002**	72
					Host: *N. pulchra*	0.19 ± 0.03	–6.79	**<0.0001**	
					Total biomass	1.21 ± 0.06	21.50	**<0.0001**	
		Time to flower	GLMM‐CoxPH	Poisson	N: high	–0.09 ± 0.24	0.35	0.730	78
					Host: *N. pulchra*	–0.08 ± 0.24	–0.35	0.720	
					Total biomass	2.35 ± 0.52	4.54	**<0.0001**	
	*Nasella* spp.	Total biomass	LM	Gaussian	N: high	–0.17 ± 0.13	–1.32	0.190	78
					Host: *N. pulchra*	0.18 ± 0.13	1.41	0.160	
		Total biomass	LM	Gaussian	Total biomass (*C. exserta*)	–0.88 ± 0.27	–3.18	**0.002**	
		Aboveground biomass	LM	Gaussian	N: high	–0.06 ± 0.08	–0.81	0.420	78
					Host: *N. pulchra*	0.01 ± 0.08	0.10	0.920	
		Belowground biomass	LMM	Gaussian	N: high	–0.11 ± 0.06	–1.74	0.090	78
					Host: *N. pulchra*	0.18 ± 0.06	2.83	**0.006**	

For all *C. exserta*, the number of haustoria, a proxy for strength of parasitism, was best predicted by host presence, N level, parasite total biomass and the interaction of biomass and N level. These results suggest that haustoria formed more readily with increasing biomass at low N (*R* ^2^ = 0.30, slope = 231) compared to when grown at elevated N (*R* ^2^ = 0.10, slope = 93; Table 1; Figure 2B). For host–parasite pairs, total number of haustoria was best predicted by species identity and N level and covaried with total biomass (Table 2). Overall, haustoria developed along a size gradient likely linked to time since formation along the root, with some individuals generating high numbers of total nodules (>300; Figure 2B; Appendix S1: Figure S2; Appendix S2: Table S1). The largest average number of haustoria (>90 per plant) developed in the low N treatment when paired with a host.

Elevated N reduced the total haustoria formed and the number across each of the two largest size classes (GLMM, C size class N level: –0.60 0.04, *z*_106_ = –16.97, *P* < 0.001; GLMM, D size class N level: –0.17 0.06, *z*_106_ = –3.08, *P* = 0.002; Appendix S2: Table S1). Notably, lack of a host did not prevent haustoria formation because nodules formed on overlapping roots of the same plant, but elevated N reduced this occurrence by ~50% to very few total haustoria. Among hosts, more haustoria formed on *N. lepida* than *N. pulchra* across N levels in total (GLMM, *z*_110_ = 3.3, *P* = 0.002) and for each size class (GLMM c: *z*_110_ = 2.5, *P* = 0.03; d: *z*_110_ = 3.0, *P* = 0.009; Appendix S2: Table S1). Overall, haustoria numbers increased with all measures of biomass, suggesting increased performance during active parasitism. For those grown without hosts, the number of haustoria was positively correlated with total biomass (*R* ^2^ = 0.41, *z*_35_ = 9.06, *P* < 0.001). Although some individuals achieved high biomass with many haustoria (e.g., 0.89 g with 463 haustoria; Appendix S2: Table S3), our outlier analysis did not detect any points above the expected hat‐value threshold (0.071). Seven haustoria counts influenced the model (Cook's distance >1), but these occurred across treatments, and iteratively removing each point or running the model with all points removed did not change the overall effects.

Flowering time was best predicted by host presence, N level, parasite total biomass, and the interaction of biomass and N level, suggesting time to the event decreased more rapidly with increasing biomass when no host was attached (Figure 2C, R^2^ = 0.31, *m* = –30.0) compared to when a host was present (*R* ^2^ = 0.25, *m* = –17.4). Hazard analysis indicated a hazard ratio (HR) of 554 for total biomass and HR of 0.03 for the interaction of host presence and total biomass. Thus, larger biomass greatly increased the likelihood of flowering, but lack of host attachment reduced total biomass influence on time to flowering. Overall, time to flowering was negatively related to total biomass with larger plants flowering earlier (Table 1, Figure 2C). These larger plants were those that were paired with hosts and grew more biomass aboveground (and in total; Table 1; Appendix S2, Table S1). Neither host presence (*t*_112_ = 0.82, *P* = 0.41) nor N level (*t*_112_ = –0.59, *P* = 0.55) influenced total stem height, but there was a marginal interaction (*t*_112_ = 2.02, *P* = 0.05). Stem number was not predicted by host presence or N level.

In the in vitro root growth bioassays, N addition reduced overall root length, but species pairing interacted with N addition to alter root behavior (Table 1; Appendix S2: Table S2). Both elevated and high additions of N reduced total root length compared to zero N, but *C. exserta* roots were longer when adjacent to *N. lepida* versus *N. pulchra* (Table 1). Root behavior (turn vs no turn) depended on N level ($\chi_{2}^{2}$ = 13.64, *P* = 0.001) with both elevated and high N resulting in reduced turn behavior ($\chi_{1}^{2}$ = 7.5, *P* = 0.006; $\chi_{1}^{2}$ = 11.3, *P* = 0.0008). Turn behavior also depended on focal seed identity ($\chi_{2}^{2}$ = 8.5, *P* = 0.014) with increased turns when adjacent to *N. lepida* compared to *N. pulchra* ($\chi_{1}^{2}\;$= 8.1, *P* = 0.004). *Nassella pulchra* tended to reduce turn behavior compared to *C. exserta* growing adjacent to another *C. exserta* ($\chi_{1}^{2}$ = 3.4, *P* = 0.06). Overall, focal seed attractiveness was not affected by N level but was greatest for *N. lepida* (0.32 ± 0.14 cm, *t*_143_ = 2.2, *P* = 0.03), negligible for *N. pulchra* (–0.04 ± 0.16 cm, *t*_143_ = –0.24, *P* = 0.8), and repulsive when *C. exserta* grew adjacent to itself (–0.58 ± 0.15 cm, *t*_143_ = –4.0 *P* < 0.001; Table 1; Appendix S2: Table S2).

## DISCUSSION

Nitrogen deposition from anthropogenic emissions alters species interactions and stability, especially in resource‐limited environments and for sensitive species (Stevens et al., 2004; Bowman et al., 2018). Here we showed that the common annual root hemiparasite, *C. exserta*, is sensitive to N amendment at urban deposition levels, and subsequently altered root growth, connectivity to host, and performance. These results extend the hypothesis that N represses haustoria formation to another hemiparasitic species; however, we also showed that current rates of N deposition have consequences for parasitic efficacy including overall growth and time to flowering, potentially disrupting species interactions.

How does N alter hemiparasitic behavior and growth? Hemiparasites largely evolved nutrient acquisition strategies because of low‐nutrient environments and as such, their physiological functions can be linked to host N content (Yuan et al., 2023; Zhang et al., 2023; Frederica and Irving, 2024). Because N is typically limiting for all plants, increasing N often stimulates host biomass and can either reduce (Liu et al., 2017) or stimulate (Frederica and Irving, 2024) hemiparasitic growth. The directionality of the interaction depends on several factors, including whether the host plant responds to enhanced N and the manner of connectivity (stem parasites lack roots that would otherwise take up some environmental N). We used extant soil N concentrations commonly found within our species’ ranges as our baseline and added N up to levels occurring at regional locations experiencing recent N deposition (Allen et al., 2014). That the growth of neither host species was stimulated by the increase in N suggests that typical soils not experiencing deposition may be at or near saturation for these species. *Nassella*/*Stipa* species are common to habitats that often experience greater levels of aridity and temperature. Some species (*S. grandis* and *S. krylovii*) from grasslands in northern China achieve similar biomass in soils containing different N levels, suggesting different N‐use efficiency mechanisms (Yuan et al., 2005). When a gradient of N amendment was applied at levels similar to and above this study (0–500 kg ha^–1^), *S. krylovii* did not increase in biomass although species richness declined across the community (He et al., 2016). Yet another species (*S. purpurea*) from an alpine steppe increased in biomass when fertilized with N at 20× the annual depositional rate (Liu et al., 2011; Yu et al., 2023). Thus, variation in N application rates and lack of direct species comparisons support our findings that North American *Nassella*/*Stipa* species may have a high N‐use efficiency, but more study across species and extreme N levels is needed.

A lack of N stimulation in biomass is not uncommon and appears to depend on life history and relative amount of N applied. For example, both N‐fixing and non‐fixing species challenged similarly with a stem hemiparasite and N amendment (~44 kg ha^–1^) did not increase in biomass after fertilization (Cirocco et al., 2017), linking other factors such as host life history (e.g., N fixation via *Rhizobia)* to N stimulation in photosynthesis and biomass. Similarly, mild N addition (17.3 kg ha^–1^) to agar bioassays did not increase root growth of a grass host, but did increase that of a legume host (Sandner et al., 2022). In this context, the lack of stimulation we observed may diminish the host ability to compensate for the negative effects of parasitism or reduce its competitive ability against other invasive plants that respond to increased N availability (Fenn et al., 2003).

By contrast, adding N at deposition levels altered *C. exserta* performance with some dependence on whether it was connected to a host. For example, elevated N increased *C. exserta* biomass in shoots and roots but only when paired with a host. There was no effect on biomass when grown alone, suggesting the stimulation by N was mediated by host N/uptake and connectivity, similar to observations from both stem (Zhang et al., 2023) and root hemiparasites (Jiang et al., 2008; Matthies, 2021). Because biomass increased only during attachment, other factors such as heterotrophic carbon gain may coincide with N theft and contribute to the observed effect (Těšitel et al., 2011). This N‐driven increase in *C. exserta* biomass may also reduce the ability for the *Nasella* hosts to use any additional N that is taken up, further diminishing their competitive ability and resulting in the observed effects that N did not stimulate *Nassella* species growth. These increases in *C. exserta* biomass contrast with the reduced haustoria number under elevated N levels whether grown alone or paired with a host. Ultimately, the combined direct effects of elevated N on *C. exserta* and indirect effects mediated by host attachment resulted in larger plants flowering earlier with a lack of hosts reducing time to flowering relative to size. If we assume size is a proxy for fitness, these effects likely reduce overall recruitment and may lead to reduced presence within a community experiencing deposition over time. The effects of N levels on hemiparasites are mixed in the literature, largely resulting from the amount of N applied and variation in experimental design. When high N (100 kg ha^–1^) levels were applied to a community containing *R. minor*, its percentage cover declined each year over 5 years (Hejcman et al., 2011). For *R. alectorolophus*, biomass increased along a gradient of increasing N (up to 64 kg ha^–1^) even if hosts showed species‐specific increases (Korell et al., 2020). In more complex evaluations of N, a supply of inorganic N via fertilization increased biomass more than N naturally sourced from *Rhizobia*; however, the total N available in the host plant was also lower in inoculation‐only treatments (Jiang et al., 2008). Taken together, these results paint a complex picture where N source (organic vs. inorganic), absolute levels of N available, and host responsiveness (i.e., NUE) determine how parasites respond to increased N in their environment when attached to hosts. At a community level, these patterns in biomass reduction and predicted reductions in recruitment may help explain the anecdotal evidence that citizen observations of *C. exserta* (via inaturalist.org) are diminished similar to how other N‐impacted symbionts (e.g., mycorrhizae) and N‐sensitive plant species show decline in regions of high N deposition (Allen et al., 2016; Simkin et al., 2016).

Root hemiparasites typically grow in search of their hosts and are often directed by root exudates (Yoder, 2001; Li et al., 2022; Sandner et al., 2022). We tested how N addition altered the growth of *C. exserta* toward a newly germinating host seed or itself and found total root growth (length) decreased with increasing N for all species, in support of an expectation of reduced searching when nutrient supply is ample. We also found elevated N reduced root behavior (turning) toward hosts or away from other *C. exserta* germinants. While many parasitic plants are generalists, some species show host preferences (Runyon et al., 2006; Li et al., 2022; Sandner et al., 2022). We found *C. exserta* grew more when adjacent to, turned more toward, and thus had overall higher attraction to *N. lepida* than *N. pulchra* and even exhibited repulsion to conspecifics. In combination with the results that more haustoria grew on *N. lepida* and *N. pulchra* had greater root biomass, there may be a host preference for *N. lepida*—perhaps mediated by some level of resistance in *N. pulchra*—that may be relevant to conservation or restoration management. While these differential shifts in root behavior may be mediated by a chemical signal such as strigalactones and other haustorium‐inducing factors diffusing from the host (Xie et al., 2010) or an ability to sense self and likely avoid intraspecific competition (Nabity et al., 2021), both greenhouse and in vitro assays demonstrated an effect of N on hemiparasite performance. Thus, additional studies on this and other *Castilleja* species may help isolate the cues used by this genus to locate hosts within the soil and confirm how broadly and what loads of excess N interact with these cues to reduce host seeking abilities.

What are the cascading effects, if any on the plant community? Plant parasites exert cascading effects within their communities by altering plant diversity and productivity (Bardgett et al., 2006) or evenness (Hodžić et al., 2022). However, species interactions often change as climatic shifts result in new geographic ranges and overlap, and as anthropogenic drivers of climate change like N emissions directly alter plant function. Enhanced N directly altered performance and parasitism by *C. exserta*, and larger plants flowered earlier (Figure 2C). The combination of these factors indicated *C. exserta* will flower slightly sooner when grown without hosts than those with hosts, but larger biomass largely drives the onset of flowering. Thus, we might predict the direct effect of N to reduce haustoria will result in an indirect effect on pollination. For example, if warming temperatures alter plant phenology to earlier flowering (Fitter and Fitter, 2002; Rafferty et al., 2020), but a lack of parasitic connections under N deposition reduce biomass and delay flowering, then these may counteract to maintain flowering time in areas experiencing deposition. But in these same areas, if pollinators shift in response to warming but their host plants do not, then synchrony may be reduced (Manincor et al., 2023). Alternately, if a parasite succeeds in attaching to a host in a site experiencing elevated N, the stimulation in parasite biomass may lead to earlier flowering and thus maintain synchrony. How these patterns alter *C. exserta* recruitment or pollinator persistence across populations in depositional and natural N environments requires additional study.

## CONCLUSIONS

Plant parasites serve fundamental roles in ecosystems and evolved their habit under specific environmental context. Anthropogenic N deposition alters this context and, in modifying plant–parasite interactions, may induce cascading effects on competition, facilitation, and related trophic interactions, such as pollination. By assessing these cascading effects at the community level, we can begin to understand the broader consequences of loss in parasitic interactions.

## AUTHOR CONTRIBUTIONS

J.T. and P.D.N. conceived and designed the experiment. J.T. collected the data. J.T. and P.D.N. analyzed the data and drafted the manuscript. P.D.N. revised the manuscript and secured funding.

## Supporting information


**Appendix S1.** Experimental design and haustoria size classes.
**Figure S1.** (a) Experimental design showing species grown alone (C, L, or P) or paired (C + L and C + P) and (b) example of a branched stem for *C. exserta* growing adjacent to *N. lepida*.
**Figure S2.** Size classes of haustoria of *Castilleja exserta*.
**Figure S3.** Graphical depiction of experimental design (A) and seed plates (multiple seeds per plate) showing variation in seedling sizes for *Castilleja exserta* relative to *Nassella* spp. (B) or another *C. exserta* seed (C).

**Appendix S2.** Statistics for greenhouse and in vitro assays.
**Table S1.** Performance metrics of *Castilleja exserta* and *Nassella* host species grown alone or paired at different N levels.
**Table S2.** Root performance metrics during the germination assay for *Castilleja exserta* grown adjacent to itself or a *Nassella* host species. Length of root growing toward (t) or away (a) from the focal seed was measured either before the first root turn (PT) or after the turn (AT) and summed for attraction (PTt+ATt) – (PTa+ATa).
**Table S3.** Outlier values tested for leverage, as described in the methods, were found across host, species, and N treatments.

## Data Availability

All raw data are publicly available online at Zenodo: https://doi.org/10.5281/zenodo.13958648.
