# Supplementary material for: Nitrogen enrichment reduces parasitism in an annual hemiparasite
Source: Am J Bot. 2025 Sep 18;112(9):e70101. doi: 10.1002/ajb2.70101 (PMC12464460; doi:10.1002/ajb2.70101)
Supplement: Supplementary file 1 — Appendix S1. Experimental design and haustoria size classes. Figure S1. (a) Experimental design showing species grown alone (C, L, or P) or paired (C + L and C + P) and (b) example of a branched stem for C. exserta growing adjacent to N. lepida. Figure S2. Size classes of haustoria of Castilleja exserta. Figure S3. Graphical depiction of experimental design (A) and seed plates (multiple seeds per plate) showing variation in seedling sizes for Castilleja exserta relative to Nassella spp. (B) or another C. exserta seed (C). [file AJB2-112-e70101-s002.docx]

Appendix S1. Experimental design and haustoria size classes.


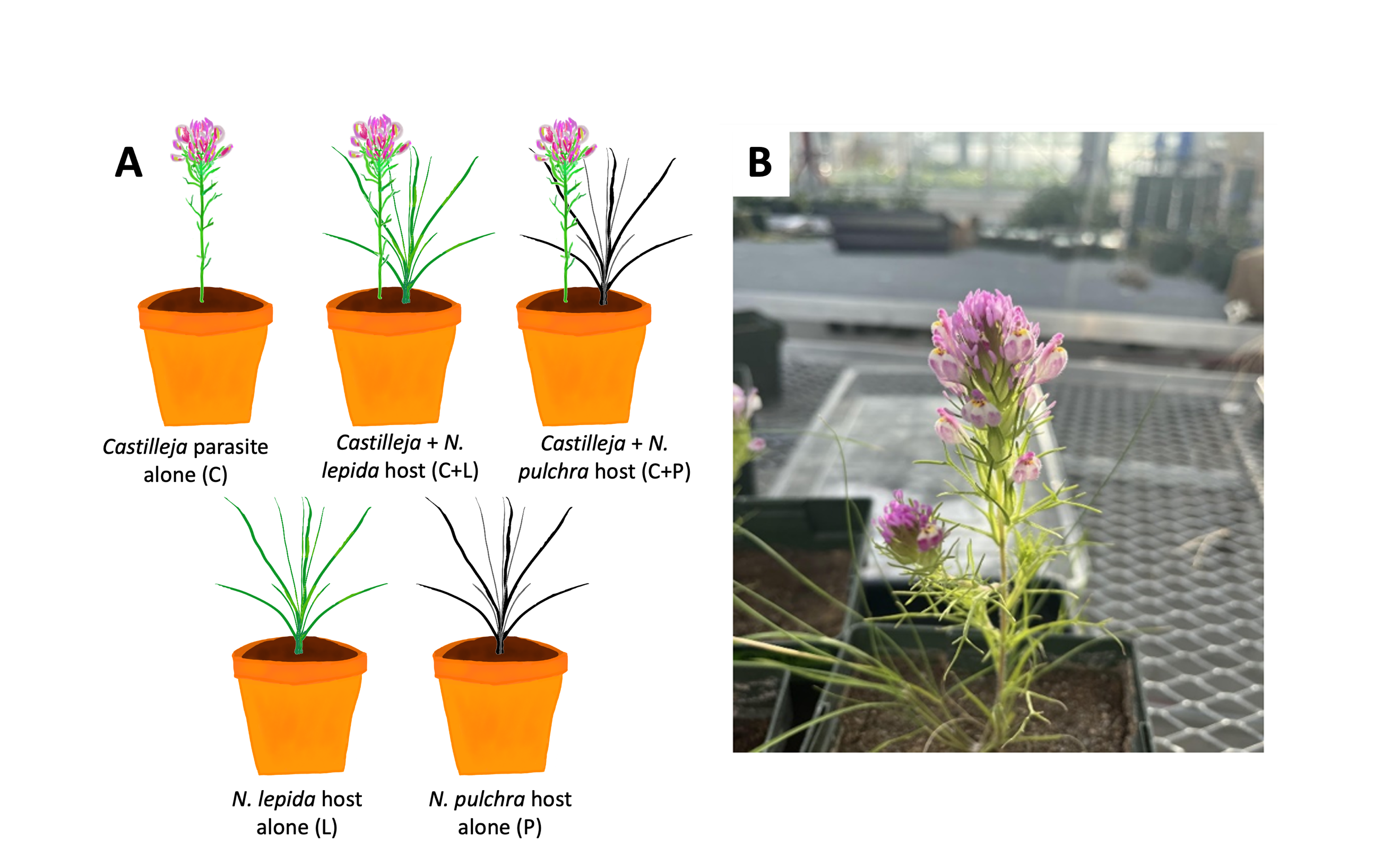


Figure S1. (a) Experimental design showing species grown alone (C, L or P) or paired (C+L and C+P), and (b) branched stem for *Castilleja exserta* growing adjacent *Nasella lepida*.


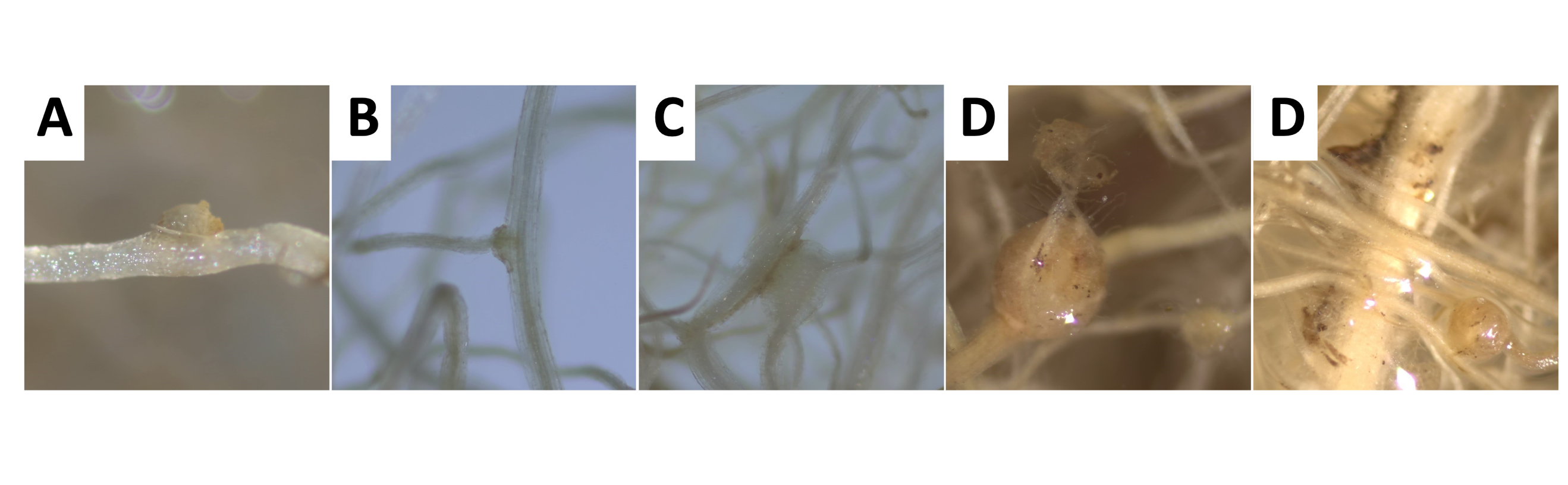


Figure S2. New haustoria of *Castilleja exserta* formed throughout the experiment, resulting in various size classes. Sizes A and B were more numerous but not counted because root swellings and a physical connection to another root could not be differentiated. Sizes C and D show visible connections (xylem bridge to another root) that discolor to orange with age/size. Classes were defined as (A) pre-haustorial swelling with no connection, (B) haustorial swelling with root overlap (but hard to distinguish from lateral root), (C) haustorial swelling with visible xylem bridge to another root, and (D) large swelling, often orangish with haustorial connection.


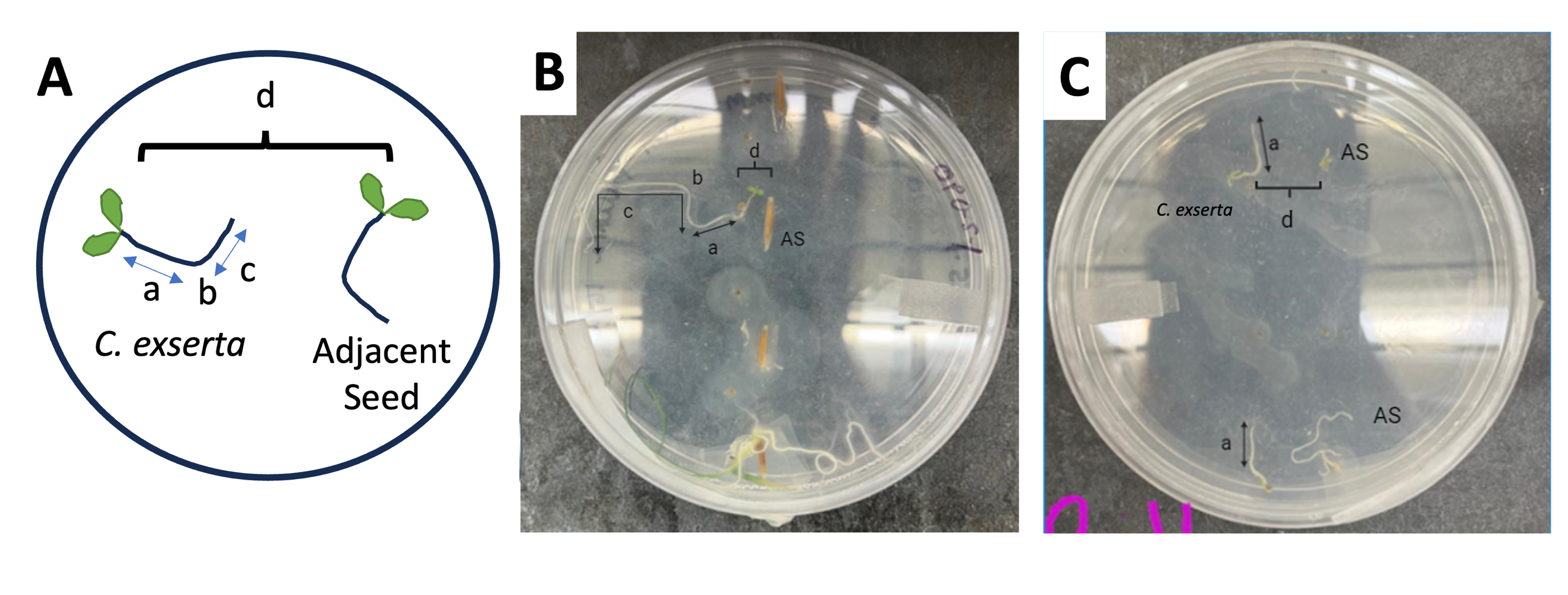


Figure S3. Graphical depiction of experimental design (A) and seed plates (multiple seeds per plate) showing variation in seed growth for *Castilleja exserta* growing relative to *Nassella* spp. (B) or another *C. exserta* seed (C). Adjacent seeds (AS) were plated at a distance (d) between *C. exserta*; the AS and metrics measured included (a) preturn radical extension toward (+) or away (-) AS, (b) point of root turn if present, and (c) after turn root length toward or away from the AS.
