# Supplementary material for: Nitrogen enrichment reduces parasitism in an annual hemiparasite
Source: Am J Bot. 2025 Sep 18;112(9):e70101. doi: 10.1002/ajb2.70101 (PMC12464460; doi:10.1002/ajb2.70101)
Supplement: Supplementary file 2 — Appendix S2. Statistics for greenhouse and in vitro assays. Table S1. Performance metrics of Castilleja exserta and Nassella host species grown alone or paired at different N levels. Table S2. Root performance metrics during the germination assay for Castilleja exserta grown adjacent to itself or a Nassella host species. Length of root growing toward (t) or away (a) from the focal seed was measured either before the first root turn (PT) or after the turn (AT) and summed for attraction (PTt+ATt) – (PTa+ATa). Table S3. Outlier values tested for leverage, as described in the methods, were found across host, species, and N treatments. [file AJB2-112-e70101-s001.docx]

**Appendix S2.** Statistics for greenhouse and in vitro assays.

**Table S1.** Performance metrics of *Castilleja exserta* and *Nassella* host species grown alone or paired at different N levels. Haustoria types include total and per size classes (C and D defined in methods).

| **Low N** |  | **Biomass (g)** | | | **Haustoria counts** | | | **Time (d) to** | **Stem** | **Stem** |
| --- | --- | --- | --- | --- | --- | --- | --- | --- | --- | --- |
| **Alone** | ***N*** | **Total ± SE** | **aboveground ± SE** | **Belowground ± SE** | **Total ± SE** | **C type ± SE** | **D type ± SE** | **flower ± SE** | **height (cm)** | **number** |
| *C. exserta* | 18 | 0.289±0.035 | 0.275±0.031 | 0.016±0.003 | 10±3 | 7±2 | 3±1 | 106±1 | 24.8±1.6 | 1.1±0.1 |
| *N. lepida* | 20 | 2.18±0.15 | 1.276±0.1 | 0.904±0.068 |  |  |  |  |  |  |
| *N. pulchra* | 19 | 2.265±0.191 | 1.224±0.121 | 1.041±0.083 |  |  |  |  |  |  |
| **Paired** |  |  |  |  |  |  |  |  |  |  |
| *C. exserta* (+NL) | 20 | 0.375±0.052 | 0.363±0.051 | 0.013±0.002 | 94±18 | 65±10 | 28±10 | 109±2 | 28.6±2.1 | 1.3±0.1 |
| *C. exserta* (+NP) | 18 | 0.349±0.047 | 0.337±0.045 | 0.012±0.002 | 90±24 | 72±21 | 18±4 | 110±2 | 25.5±1.7 | 1.1±0.1 |
| *N. lepida* | 20 | 1.198±0.112 | 0.643±0.08 | 0.555±0.047 |  |  |  |  |  |  |
| *N. pulchra* | 20 | 1.468±0.154 | 0.664±0.072 | 0.804±0.088 |  |  |  |  |  |  |
| **Elevated N** |  |  |  |  |  |  |  |  |  |  |
| **Alone** |  |  |  |  |  |  |  |  |  |  |
| *C. exserta* | 20 | 0.255±0.034 | 0.241±0.033 | 0.014±0.003 | 5±1 | 4±1 | 1±0 | 108±2 | 22.8±2.0 | 1.1±0.1 |
| *N. lepida* | 20 | 2.293±0.232 | 1.297±0.139 | 0.996±0.106 |  |  |  |  |  |  |
| *N. pulchra* | 20 | 2.26±0.178 | 1.192±0.102 | 1.068±0.085 |  |  |  |  |  |  |
| **Paired** |  |  |  |  |  |  |  |  |  |  |
| *C. exserta* (+NL) | 18 | 0.446±0.045 | 0.449±0.043 | 0.016±0.002 | 70±21 | 52±19 | 18±3 | 106±1 | 31.9±2.1 | 1.3±0.1 |
| *C. exserta* (+NP) | 19 | 0.51±0.062 | 0.479±0.06 | 0.018±0.003 | 61±24 | 40±10 | 21±5 | 107±2 | 34.7±3.7 | 1.5±0.2 |
| *N. lepida* | 18 | 1.107±0.13 | 0.591±0.095 | 0.516±0.047 |  |  |  |  |  |  |
| *N. pulchra* | 20 | 1.209±0.128 | 0.587±0.072 | 0.622±0.069 |  |  |  |  |  |  |

Table S2. Root performance metrics during the germination assay for *Castilleja exserta* grown adjacent to itself or a *Nassella* host species for each N level. Root length toward (t) or away (a) from the focal seed was measured either before the first root turn (PT) or after the turn (AT) and summed to yield “attraction” (PTt+ATt) or (PTa+ATa).

|  |  | **Length (cm)** | | | | |  |  |
| --- | --- | --- | --- | --- | --- | --- | --- | --- |
| **Paired with itself** | ***N*** | **Total** | **PT-t** | **PT-a** | **AT-t** | **AT-a** | **Attraction** | **Turned yes(no)** |
| **0 mM N** | 27 | 1.677±0.207 | 0.228±0.067 | 0.824±0.207 | 0.195±0.087 | 0.43±0.118 | -0.83±0.256 | 17(10) |
| **10 mM N** | 7 | 0.95±0.285 | 0.14±0.084 | 0.534±0.316 | 0.026±0.026 | 0.25±0.19 | -0.61±0.376 | 3(4) |
| **25 mM N** | 16 | 0.878±0.269 | 0.355±0.197 | 0.217±0.054 | 0.013±0.013 | 0.294±0.237 | -0.14±0.329 | 3(13) |
| **Paired with *N. lepida*** |  |  |  |  |  |  |  |  |
| **0 mM N** | 22 | 1.77±0.281 | 0.294±0.063 | 0.194±0.049 | 0.846±0.193 | 0.437±0.15 | 0.508±0.229 | 17(5) |
| **10 mM N** | 19 | 1.115±0.188 | 0.314±0.07 | 0.293±0.109 | 0.385±0.154 | 0.124±0.055 | 0.282±0.237 | 11(8) |
| **25 mM N** | 12 | 0.779±0.179 | 0.196±0.085 | 0.216±0.062 | 0.208±0.096 | 0.159±0.091 | 0.029±0.137 | 6(6) |
| **Paired with *N. pulchra*** | |  |  |  |  |  |  |  |
| **0 mM N** | 16 | 1.109±0.222 | 0.203±0.054 | 0.2±0.066 | 0.134±0.055 | 0.573±0.194 | -0.43±0.201 | 9(7) |
| **10 mM N** | 15 | 0.637±0.132 | 0.356±0.125 | 0.217±0.1 | 0.065±0.051 | 0±0 | 0.204±0.179 | 2(13) |
| **25 mM N** | 12 | 0.75±0.216 | 0.259±0.062 | 0.135±0.047 | 0.211±0.154 | 0.145±0.068 | 0.19±0.149 | 4(8) |

**Table S3.** Outlier values tested for leverage, as described in the methods, were found across host, species, and N treatments.

| **Host** | **Species pair ID** | **N level** | **Total biomass (g)** | **Total haustoria** | **Residuals** | **Hat distance** | **Cook’s distance** | **Sample line from data set** | |
| --- | --- | --- | --- | --- | --- | --- | --- | --- | --- |
| yes | *N. lepida* | high | 0.2961 | 397 | 27.759279 | 0.02702703 | 12.0473477 | 73 |  |
| yes | *N. pulchra* | low | 0.8885 | 463 | 27.517259 | 0.02702703 | 10.7394164 | 93 |  |
| yes | *N. lepida* | low | 0.266 | 327 | 19.00484 | 0.02702703 | 4.3136416 | 41 |  |
| yes | *N. pulchra* | high | 0.5497 | 228 | 15.658197 | 0.02702703 | 2.9000361 | 106 |  |
| yes | *N. pulchra* | high | 0.8399 | 220 | 15.014558 | 0.02702703 | 2.6219962 | 107 |  |
| no | none | low | 0.4373 | 50 | 8.898896 | 0.05555556 | 2.410185 | 3 |  |
| no | none | high | 0.5389 | 25 | 6.10503 | 0.05263158 | 1.0366775 | 32 |  |
